# Effect of Carboxymethylation on the Rheological Properties of Hyaluronan

**DOI:** 10.1371/journal.pone.0162849

**Published:** 2016-09-09

**Authors:** Rian J. Wendling, Amanda M. Christensen, Arthur D. Quast, Sarah K. Atzet, Brenda K. Mann

**Affiliations:** 1 Department of Bioengineering, University of Utah, Salt Lake City, Utah, United States of America; 2 SentrX Animal Care, Inc., Salt Lake City, Utah, United States of America; 3 Department of Chemistry, University of Utah, Salt Lake City, Utah, United States of America; University of Michigan, UNITED STATES

## Abstract

Chemical modifications made to hyaluronan to enable covalent crosslinking to form a hydrogel or to attach other molecules may alter the physical properties as well, which have physiological importance. Here we created carboxymethyl hyaluronan (CMHA) with varied degree of modification and investigated the effect on the viscosity of CMHA solutions. Viscosity decreased initially as modification increased, with a minimum viscosity for about 30–40% modification. This was followed by an increase in viscosity around 45–50% modification. The pH of the solution had a variable effect on viscosity, depending on the degree of carboxymethyl modification and buffer. The presence of phosphates in the buffer led to decreased viscosity. We also compared large-scale production lots of CMHA to lab-scale and found that large-scale required extended reaction times to achieve the same degree of modification. Finally, thiolated CMHA was disulfide crosslinked to create hydrogels with increased viscosity and shear-thinning aspects compared to CMHA solutions.

## Introduction

Hyaluronan, or hyaluronic acid, (HA) is a physiologically important biopolymer that, depending on the tissue, provides water homeostasis, joint lubrication, shock absorption, and stabilization of the extracellular matrix (ECM) [1–3]. These functions, in part, derive from its chemical structure, and it is surmised that a combination of hydrogen bonding and electrostatic repulsion involving the hydroxyl, carboxyl, and acetamido groups lead to its viscoelastic properties in solution [4,5]. It is also known that these properties depend on the molecular weight of the HA [6]. For instance, a solution of HA will exhibit shear-thinning, but only above a sufficiently high concentration and/or molecular weight.

Due to the beneficial properties of HA, it has become an important biomaterial used for a variety of medical products. These products cover a range of applications, including osteoarthritis [7,8], keratoconjunctivitis sicca (dry eye) [9], wound healing [10,11], post-surgical adhesion prevention [12], drug delivery [13], and dermal fillers [14, 15]. Many of these products utilize a simple solution of HA; however, when introduced into the body, the HA is readily degraded. Although the HA fragments may provide a cellular benefit through interaction with receptors on the cell surface, it quickly loses the physical properties that provide the additional benefit to the ECM. In order to extend the residence time in the body, exogenously administered HA can be covalently crosslinked together to form a much larger network with a slower degradation rate than a simple HA solution.

Covalent crosslinking is often accomplished by first modifying the HA to introduce a more reactive site, followed by crosslinking at the new reactive site. Modification of the HA typically occurs at either the hydroxyl site or the carboxyl site. Examples of modification at the hydroxyl site include sulfation, esterification (with methacrylic anhydride), etherification (with epoxide or vinyl sulfone), and periodate oxidation (reductive coupling with amines). Examples of modification at the carboxyl site include esterification and carbodiimide-mediated coupling of sulfo(NHS), hydrazides, and methacrylamide. Review papers describing these modifications and others for creating HA-based biomaterials have been previously published [16,17].

**Fig 1:**
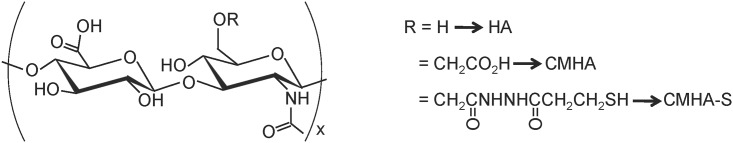
General structure of hyaluronan. Modifications at the R group are indicated to generate CMHA and CMHA-S.

In the past few years, we have made extensive use of a two-step modification of HA to produce thiolated carboxymethylHA (CMHA-S). This is achieved by first introducing additional carboxyl groups at some of the hydroxyl sites, followed by carbodiimide-mediated coupling of a hydrazide to carboxyl groups to provide free thiol groups [18] (Fig 1). These thiol groups are then used to covalently crosslink the CMHA-S through either oxidative disulfide crosslinking or using an external crosslinker that has thiol-reactive groups (such as an acrylate, bromoacetamide, or maleimide) [19,20]. Although we have extensively investigated the CMHA-S and its physical and biochemical properties once crosslinked, we have not examined the properties of the CMHA (prior to thiolation), particularly in regards to how the degree of modification might impact the resultant physical properties of the biopolymer in solution.

Therefore, in order to better understand how the properties of the HA might change upon modification of the hydroxyl groups to provide additional carboxyl groups to the HA, we have produced CMHA with varying degrees of CM modification and examined the effect of the modification on the rheological properties of the CMHA in solution. The effect of concentration, buffer type, and pH on the rheological properties were all investigated. Additionally, we compared the modification process used on the lab-scale versus production-scale to assess the impact on both CM modification and molecular weight. Finally, we compared the rheological properties of a solution of CMHA to crosslinked CMHA-S with three different degrees of thiolation. With a better understanding of how a reduction in hydroxyls and a corresponding increase in carboxyls affects the overall physical properties, we may be able to use this information to produce modified HA that allows for desired covalent crosslinking without detracting from the beneficial physical properties of the HA itself.

## Materials and Methods

### CMHA synthesis

Carboxymethyl hyaluronic acid (CMHA) was synthesized as previously described [18] with a few modifications. Briefly, medical device-grade HA (Novozymes Biopolymers, Inc., Bagsvaerd, Denmark) was dissolved in 45% NaOH and stirred at room temperature for 2.5 hours to deprotonate the HA. The deprotonated HA was then reacted with chloroacetic acid in isopropanol for 1 hour, followed by precipitation for 30 minutes. The liquid was decanted, and the resultant carboxymethyl HA (CMHA) was dissolved in deionized (DI) water. The pH was adjusted to 7.0 and the CMHA was purified against DI water by either dialysis or tangential flow filtration (TFF).

For small lots (starting with 5g of HA), the MW of the HA was 900 kDa; for production lots (starting with 300-400g of HA), the MW of the HA was 800 kDa. To vary the degree of carboxyl modification in the small lots, the amount of chloroacetic acid (0-10g) and the reaction time with the chloroacetic acid (0.5–2.5 hr) were varied (see Table 1). The reaction time with chloroacetic acid in production lots was 2.5 hr. CMHA produced in small lots was purified using dialysis tubing (MWCO 3.5 kDa); CMHA from production lots was purified using TFF. Following purification, CMHA was lyophilized. MW and polydispersity (PDI) of the CMHA was determined using gel permeation chromatography and compared to an HA standard.

**Table 1:** Effect of CMHA synthesis parameters on degree of modification.

Test Lot #	HA (g) / CA (g)	Time in CA (hr)	% CM modification	MW (kDa)	PDI
Mock	NA	1	0	320.7	1.48
1	1	1	39.0	339.3	1.51
2	2	1	29.4	316.9	1.56
3	0.5	1	54.0	317.7	1.55
4	1	0.5	33.6	331.6	1.59
5	1	1.5	46.6	339.2	1.50
6	1	1.5	46.5	323.8	1.47
7	1	2	46.6	314.8	1.42
8	0.5	2	74.3	288.9	1.54
9	1	2.5	48.0	295.4	1.61
10	5	1	11.7	333.8	1.48
10b	5	1	7.4	266.3	1.83
11	10	1	5.0	307.8	1.61
11b	10	1	3.2	243.0	1.77

All test lots started with 5g HA (MW 900kDa). Test lots 10b & 11b were derived from 10 & 11, respectively, and additionally underwent HCl treatment to reduce MW after carboxyl modification. NA = not applicable as mock CMHA had no CA added.

### Determination of carboxyl modification

To determine the degree of carboxyl modification of CMHA, a modified titration assay was used [21]. Briefly, CMHA was dissolved at 10 mg/ml in DI water. Cationic exchange resin (Dowex 50WX8, 100–200 mesh, Sigma-Aldrich) was added to the solution and was mixed for 2 hrs at 37°C. The solution with resin was then filtered first through a 0.8μm filter and then through a 0.22μm filter to remove the resin. The resulting solution of acidified CMHA was then lyophilized. Dry acidified CMHA (125 mg) was dissolved in 10ml of 0.1N NaOH and diluted with 15ml of DI water. This solution was then titrated with 0.05N HCl, using phenolphthalein as an indicator. Blank titrations contained no CMHA. The degree of substitution was calculated using the following equations:

$$n_{COOH}=\left(V_{b}-V\right)\times C_{HCl}$$ (1)

where: n_COOH_ = mol. carboxyl grps

V_b_ = vol. HCl needed to titrate blank

V = vol. HCl needed to titrate sample

C_HCl_ = conc. of HCl

$$DS=\left(MW_{DSU}\times n_{COOH}\right)÷\left(m_{dry}-MW_{I}\times n_{COOH}\right)$$ (2)

where: DS = degree of substitution

MW_DSU_ = MW of an unsubstituted disaccharide unit

m_dry_ = mass of dry CMHA

MW_I_ = increase in MW due to carboxyl grp substitution

The percent carboxyl modification was then calculated *DS* × 100%. Each lot of CMHA was acidified twice separately, and the percent modification of the two runs averaged.

### FTIR analysis

FTIR spectra of neat HA, mock CMHA, and CMHA samples were collected on a Thermo Scientific Nicolet iS50 FT-IR using the manufacturer's ATR accessory, with the resolution set to 4.000 cm^-1^, and 64 scans being averaged for each sample. Background spectra were collected before each sample, and raw data was collected in % transmittance mode. The minimum % transmittance for all raw spectra occurred at 1028 cm^-1^ and was no less than 48% (0.32 absorbance units) for any sample. Data were then converted to absorbance units and normalized so that the peak at 3280 cm^-1^ was equal to unity.

### Rheological assessment

CMHA and HA were dissolved at various concentrations (10–40 mg/ml) in phosphate-buffered saline (1X PBS; Fisher Scientific), at 25 mg/ml in different buffers (DI water, 0.1X PBS, 1X PBS, 10X PBS, 0.9% saline), and at 25 mg/ml in 0.9% saline at various pHs (pH 2–10). Conductivity of the buffers with and without CMHA (25 mg/ml) was determined using a conductivity meter (Fisher Scientific). Rheological testing to determine viscosity of CMHA and HA solutions was performed as previously described [22]. G’ and G” were not determined as initial assessments indicated that for solutions of CMHA in the MW and concentration ranges used here, the viscous component always dominated (i.e., G”>>G’; data not shown). Thus, only viscosity measurements were subsequently used to assess the rheological properties. Three separate samples were prepared for each solution and the viscosity averaged for the three samples.

### CMHA-S synthesis and crosslinking

CMHA in production lots was further modified with thiol groups directly from TFF purification to produce CMHA-S as previously described [18]. Thiol modification was assessed using an Ellman assay [22]. CMHA-S was then disulfide crosslinked to produce a gel as previously described [22]. Viscosity of the crosslinked CMHA-S gel was determined as described above.

### Replicates of synthesis lots

Single lots were made for each small scale syntheses, whereas multiple lots were used in the analysis of production scale syntheses. For rheological assessment of small test lots, as mentioned in 2.4 above, three samples for each lot were prepared and the viscosity averaged. Standard deviations on these averages were always within 5% of the average, and more typically within 1%. As this average only represents variability in preparing the sample from a single lot, as well as any variability in the rheometry testing, and not lot-to-lot variability, only averages are shown in the Results. For production scale, three lots were assessed for CM modification and MW, thus providing a measure of lot-to-lot variability in the synthesis.

## Results

### Effect of synthesis parameters on % CM modification and MW

In the carboxymethylation process, the amount of chloroacetic acid (CA) used and the time of reaction with the CA were varied, and the resultant % modification and MW were determined. In general, as the amount of CA increased, there was an increase in the % modification (Table 1). There appears to be less of a dependence on the time of reaction, although significantly longer or shorter times can lead to a change in % modification. The % modification was varied from as low as 5% to as high as 74% (without subsequent HCl treatment).

**Fig 2:**
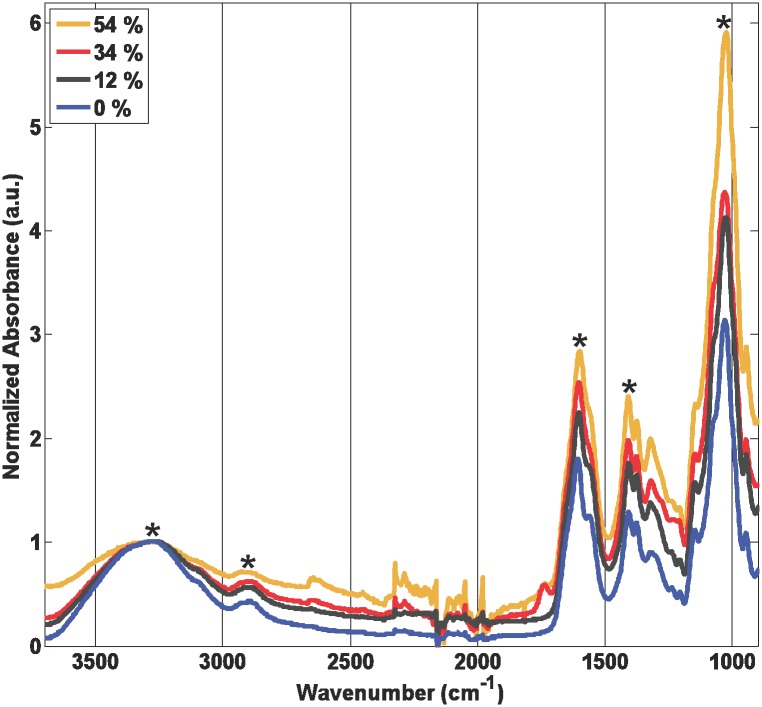
FTIR spectra of CMHA. Spectra are shown for CM modifications of 0 (mock CMHA), 12, 34, or 54%. Absorbance was normalized to the peak for the O-H & N-H stretch around 3280 cm^-1^. Assignment of peaks with asterisks is provided in the text.

The FTIR spectrum of the mock CMHA (Fig 2) showed a broad band around 3280 cm^-1^ due to O-H and N-H stretching, bands around 2900 cm^-1^ due to C-H stretching modes of both methyl and methylene groups, bands at 1600 and 1410 cm^-1^ due to the antisymmetric and symmetric stretching of carboxyl (COO) groups, respectively, and a band at 1030 cm^-1^ due to the C-O-C stretch. An FTIR spectrum of the HA used to produce the CMHAs in this study was very similar to the mock CMHA (data not shown), thus indicating that the mock CMHA has not been modified. When comparing the FTIR spectra of the mock CMHA (0% CM modification) to CM modifications of 12%, 34%, and 54%, it can be seen that the bands for the C-H stretch, the antisymmetric and symmetric carboxylate stretches, and the C-O-C stretch all increase relative to the O-H and N-H stretch as the CM modification increases (Fig 2), indicating addition of the carboxymethyl group.

Due to the sodium hydroxide treatment in the first step of the modification, required to deprotonate the HA, the MW is reduced from the HA starting MW of 900 kDa to an average of 319 ± 17 kDa, with an increase in PDI from 1.21 to 1.53 ± 0.06. It appears that very long reaction time (2.5 hr) or a combination of a large amount of CA (10 g) plus long reaction time (2 hr) may lead to further reduction in MW. Two of the CMHAs (10b & 11b) were also exposed to HCl treatment after modification to further reduce the MW. Table 1 indicates that such treatment did result in a reduced MW compared to their no HCl counterparts (10 & 11, respectively), with a further increase in PDI to 1.80 ± 0.04; however, this treatment also effectively reduced the % modification. It should also be noted that a mock CMHA was made, in which no CA was added, and resulted in 0% modification with a reduced MW similar to the other CMHAs.

The process for producing CMHA-S has been scaled up for commercial products, from starting with 5 g HA on the lab-scale to 300–400 g HA for production, and thus the lot-to-lot variability in % CM modification and resultant MW were assessed. For different products, the starting amount of HA is 300, 350, or 400 g, and results for 3 lots of each were averaged and are shown in Table 2. There was no significant difference in the % modification or MW based on the starting amount of HA. However, when comparing a small lot to a production lot, for the same weight ratio of HA to CA, the resulting % modification for the large lots is similar to using a very short reaction time (0.5 hr) in a small lot (~33%), despite the 5-fold reaction time for the large lots. Although the MW for the large lots was also lower, in general, than the small lots, the starting MW of HA for production lots was lower than for small lots.

**Table 2:** Variability in large-scale production of CMHA.

Lot type	Starting HA (g)	% CM modification	MW (kDa)	PDI
A	400	32.1 ± 1.0	279.3 ± 11.9	1.63 ± 0.02
B	350	27.3 ± 2.7	330.3 ± 37.9	1.47 ± 0.09
C	300	34.4 ± 2.3	297.3 ± 15.3	1.58 ± 0.03

The MW of the initial HA was 800 kDa. All production lots had a time in CA of 2.5 hr and HA(g)/CA(g) = 1. n = 3 lots for each lot type. Values are mean ± SD.

### Effect of % CM modification and MW on viscosity

**Fig 3:**
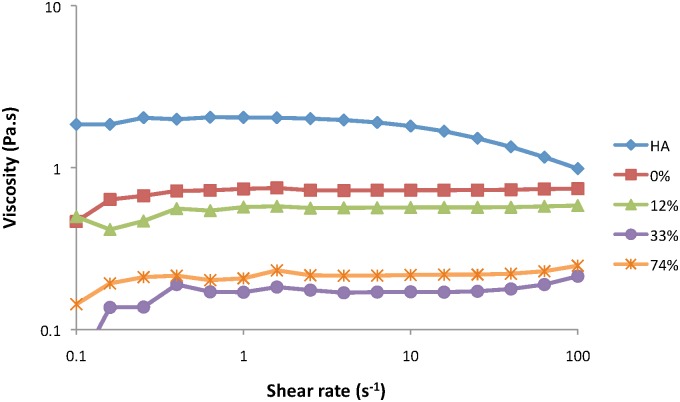
Steady-shear viscosity as a function of shear rate for solutions of HA (10 mg/ml) and CMHA (25 mg/ml) in PBS. Viscosity profiles are shown for CMHA with CM modifications of 0 (mock CMHA), 12, 33, or 74%.

Solutions of CMHA with high (74%), medium (33%), low (12%), and mock (0%) CM modification in PBS (25 mg/ml) were assessed rheologically, and the viscosity versus shear rate was compared to a solution of HA in PBS (10 mg/ml). As seen in Fig 3, the viscosity profile for HA demonstrates a typical shear thinning behavior as the shear rate increases, above a shear rate of about 5–10 s^-1^. However, this behavior is eliminated simply by reducing the MW, as observed by the flat profile for mock CMHA over the shear rate range. The profiles for all of the CMHAs are similar, although the values for the viscosity change depending on the % modification. From these profiles, it appears that the viscosity of the CMHA solution is reduced as the % modification increases from 0% to 12% to 30%, but then increases again at the highest CM modification of 74%.

**Fig 4:**
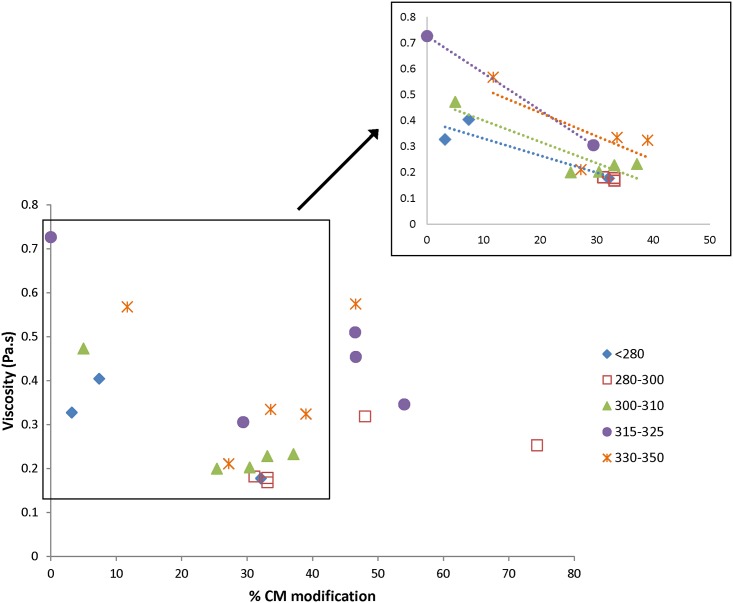
Effect of %CM modification on steady-shear viscosity. Viscosity was determined at a shear rate of 25 s^-1^ for solutions of CMHA (25 mg/ml) in PBS. Various lots of CMHA are grouped by molecular weight: <280, 280–300, 300–310, 315–325, and 330–350 kDa.

The effect of the % CM modification on viscosity could potentially be confounded by different MWs of the compounds, however, as it is known that the MW of HA affects the resulting viscosity [6]. Thus, the viscosity at a particular shear rate (25 s^-1^) was chosen to compare viscosity versus % CM modification for the various lots (Fig 4). The various lots of CMHA were broken into groups based on their MW to provide more narrow ranges of MW for comparison. When comparing within a group (i.e., a particular narrow MW range), it can be seen that the viscosity decreases as the % modification increases up to a modification of about 30–40%. This is highlighted by the trendlines shown in the inset of Fig 4. Above 40% modification, for the few samples synthesized in this study, the viscosity then increases again as the % modification approaches 45–50%. Beyond that, it appears that it may decrease again, although there were fewer samples falling within that range to provide a full analysis. Further, when comparing viscosities at a particular % modification, it is clear that as the MW of the CMHA decreases, so does the viscosity.

### Effect of CMHA concentration and buffer on viscosity

**Fig 5:**
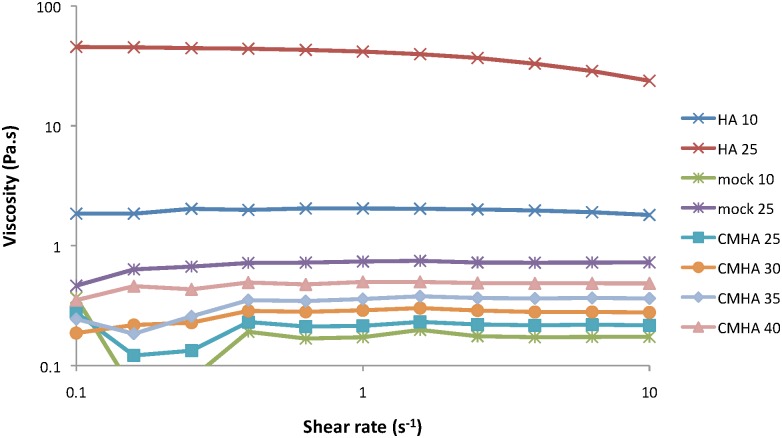
Effect of biopolymer concentration on viscosity. Steady-shear viscosity is shown as a function of shear rate for solutions of HA (10 or 25 mg/ml), mock CMHA (10 or 25 mg/ml), and CMHA (25, 30, 35, or 40 mg/ml) in PBS. Mock CMHA has 0% CM modification; CMHA has 33% CM modification.

The effect of polymer concentration and buffer on viscosity for solutions of HA, mock CMHA, and CMHA was also investigated. Fig 5 shows the viscosity versus shear rate for 2 concentrations of HA and mock CMHA, and 4 concentrations of CMHA in PBS. As expected, the viscosity decreases with decreasing polymer concentration. Table 3 provides the viscosity at a shear rate of 25 s^-1^ for HA, mock CMHA, and 2 modifications of CMHA in DI water; 0.1X, 1X, and 10X PBS; and 0.9% NaCl, the concentration used in normal saline. Solutions of the polymers in DI water have the highest viscosity, with viscosity decreasing as the PBS concentration increases. Further, the viscosity is higher for these polymers in 0.9% NaCl compared to 1X PBS, except the medium modification CMHA, indicating that the presence of phosphates reduces the viscosity. Conductivity of the buffers alone and mock CMHA or CMHA in the buffers is shown in Table 4. Buffers alone show an expected increase in conductivity with increasing salt concentration. Further, the conductivities of the solutions of CMHA are all higher than their corresponding solutions of mock CMHA.

**Table 3:** Effect of buffer on viscosity of HA and CMHA solutions.

Buffer	Medium CMHA	High CMHA	Mock CMHA	HA
DI water	0.1775	0.2590	0.9451	2.508
0.1X PBS	0.1745	0.2353	0.8158	2.295
1X PBS	0.1724	0.2185	0.7267	1.518
10X PBS	0.1737	0.2000	0.6459	1.133
0.9% NaCl	0.1714	0.2300	0.8891	1.8925

The concentration of CMHA solutions was 25 mg/ml and HA solutions was 10 mg/ml. The viscosity for each solution is at a shear rate of 25 s^-1^. Medium CMHA has a 33% modification; High CMHA has a 74% modification; Mock CMHA has 0% modification.

**Table 4:** Conductivity of solutions of CMHA.

	Conductivity (mS)		
Buffer	Buffer alone	Mock CMHA	CMHA
DI water	0.0001	5.56	19.31
0.1X PBS	1.86	6.85	24.6
1X PBS	17.10	18.70	35.8
10X PBS	113.4	114.2	124.3
0.9% NaCl	18.10	19.43	34.5

The concentration of mock CMHA (0% modification) and CMHA (33% modification) was 25 mg/ml in each of the buffers.

**Fig 6:**
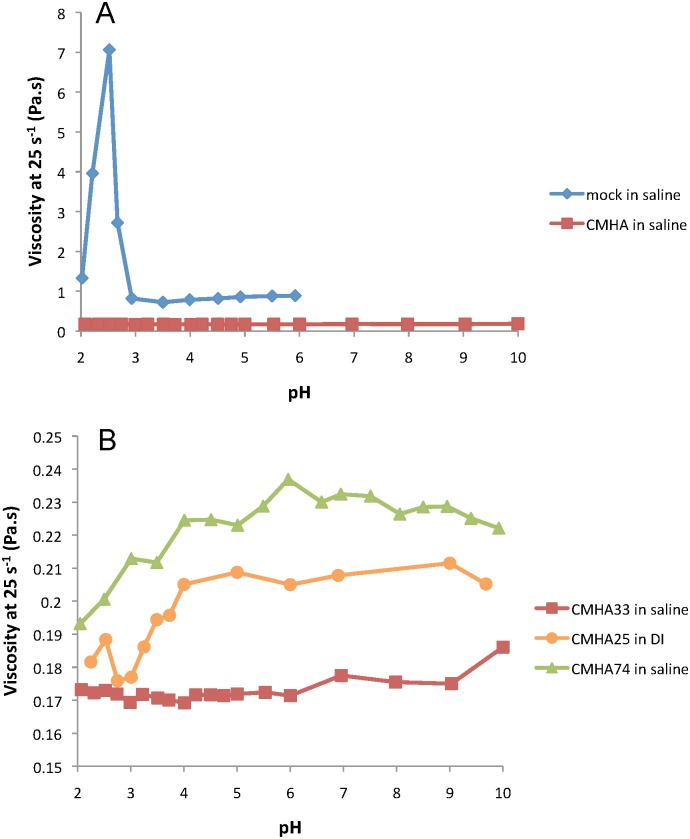
Effect of pH on steady-shear viscosity of CMHA solutions. **A:** Steady-shear viscosity at a shear rate of 25 s^-1^ for solutions of mock CMHA and CMHA (25 mg/ml) in 0.9% NaCl (saline). Mock CMHA has 0% CM modification; CMHA has 33% CM modification. **B:** Steady-shear viscosity at a shear rate of 25 s^-1^ for solutions of CMHA (25 mg/ml) in 0.9% NaCl (saline) or DI water. CMHA has 25, 33, or 74% CM modification as indicated in the legend by the number following CMHA.

Due to the charged nature of these polymers, which changes as the % modification changes, the effect of pH on viscosity for solutions of mock CMHA and CMHA was determined. Fig 6(top) shows the viscosity at a shear rate of 25 s^-1^ versus pH for both mock CMHA and CMHA in saline. The mock CMHA has a significant spike in viscosity at a pH of 2.5 whereas the CMHA has no such spike in viscosity over the pH range of 2–10. Fig 6(bottom) shows the viscosity at the same shear rate versus pH for CMHA with medium modification (25–33%) in saline or DI water compared to CMHA with high modification (74%) in saline. Although the medium modification CMHA in saline demonstrates no or little dependence of viscosity on pH, the viscosity of the same CMHA in DI water decreases with decreasing pH below about pH 4. Interestingly, the same effect is seen for high modification CMHA in saline.

### Viscosity of CMHA solutions versus crosslinked CMHA-S gels

**Fig 7:**
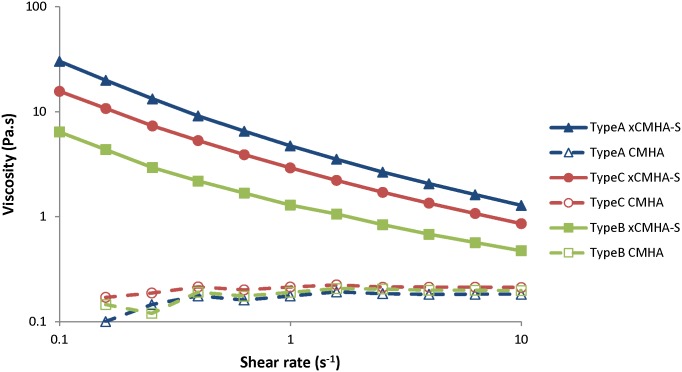
Effect of thiolation and disulfide crosslinking on viscosity of CMHA. Steady-shear viscosity as a function of shear rate for solutions of CMHA (25 mg/ml) in PBS or the corresponding thiolated and crosslinked CMHA gels (xCMHA-S). Degree of thiolation and CMHA-S concentration in the gel for each gel type is provided in Table 5.

Finally, the CMHA synthesized in production was further thiolated and crosslinked to form a gel. Table 5 provides the thiol modification and polymer concentration used to produce the three gels. The thiol modifications and polymer concentrations for these three gel types were chosen to achieve particular viscosities for their target applications. The viscosity profile of solutions of CMHA from production were then compared to their counterpart crosslinked gels (xCMHA-S). As seen in Fig 7, while the solutions of CMHA have a flat viscosity profile over the range of shear rates used here, the xCMHA-S gel shear thins over this full range of shear. As previously mentioned, the solution of HA shear thins as well, but only above a shear rate of about 5–10 s^-1^. Further, the viscosity for the xCMHA-S gel is significantly higher than corresponding solutions of CMHA. An expanded rheological characterization of gel Type B has previously been reported [22].

**Table 5:** Thiol modification of CMHA-S and concentration of CMHA-S in disulfide crosslinked gels.

Gel type	Thiol (mmol/g)	Conc. in gel (mg/ml)
A	0.39 ± 0.08	9.92 ± 0.60
B	0.23 ± 0.06	3.84 ± 0.30
C	0.15 ± 0.02	7.41 ± 0.33

Gel types correspond to the production lot types in Table 2. n = 3 lots for each gel type. Values are mean ± SD.

## Discussion

In the process of synthesizing CMHA-S, carboxymethyl groups are first added to HA that effectively displace the C6 hydroxyls on the N-acetylglucosamine, followed by the addition of thiol groups to those carboxyls. The hydroxyl that is modified is not involved in intramolecular (or likely intermolecular) hydrogen bonding of HA [5,23]. However, this may be involved in the interaction with the solvent, depending on the pH and ions present in the solvent, and therefore contribute to the overall tertiary structure of HA. By modifying it to present a carboxyl, there are three potential disruptions that could occur: it may affect intramolecular hydrogen bonding of neighboring groups, it may alter intermolecular hydrogen bonding, and it may affect solvent interaction at the site. It is also possible that the charge density of the overall molecule changes significantly, thereby affecting its properties, although this seems less likely in the high salt limit.

To investigate the effect of the degree of carboxymethylation on the physical properties of the resultant CMHA in solution, in this study we altered the degree of CM modification by changing various parameters in the synthesis process. The process also resulted in a decrease in MW of the resultant CMHA. The degree of CM modification and MW, as well as polymer concentration, buffer, and pH all influenced the viscosity of solutions of CMHA.

The degree of CM modification was determined using a titration method, and the presence of the additional CM group confirmed using FTIR. FTIR spectra of mock CMHA and HA were similar to those previously shown for HA [24–27]. Confirmation that the mock CMHA had 0% modification by both the titration method and FTIR indicate that it is effectively HA at a reduced MW from the starting HA. Spectra of CMHA with increasing %CM modification showed concomitant increases in absorbance due to vibrational modes assigned as C-H, COO, and C-O-C stretching. This is to be expected since a methylene group was added (increasing the concentration of antisymmetric and symmetric H-C-H stretches), a carboxyl group was added (increasing the concentration of antisymmetric and symmetric carboxylate stretches), and a C-O-C bridge was added (increasing the concentration of C-O-C stretches), and confirms the modification of the HA. Similar changes to FTIR spectra have been observed with carboxymethyl modification of cellulose, chitosan, and high amylose starch [28–31].

Although the MW of the resultant CMHA decreased for large amounts of CA used and/or long reaction times with the CA, the primary reduction in MW occurs during the initial reaction step in sodium hydroxide. Further, while there was some variability in MW of the CMHA among small lots and among production lots, some variability is expected simply due to polydispersity (PDI) in the starting MW of the HA, as well as the random nature of the chain breaks in the process [32]. This effectively led to an increase in PDI for the CMHA compared to the HA. Treating the CMHA with HCl was an effective method for further reduction in the MW, with a concomitant further increase in PDI; however, it also resulted in a reduction in carboxyl concentration. Thus, if a lower MW CMHA is desired, it may be best to reduce the MW of the starting HA prior to CM modification to avoid subsequently altering the CM modification.

In this study, the % CM modification influenced the viscosity of the solution, and for all MW ranges, there was an overall decrease in viscosity as the % modification increased, up to about 30–40% modification. Above this level of modification, it is difficult to determine the effect on viscosity due to the limited number of samples in this study. These data suggest that as carboxyl groups are added to the HA chain, the native interactions that lead to the physical properties typically associated with HA are disrupted. For native HA, it has been suggested that the viscoelastic properties of high MW HA in solution are due to hydrogen bonding between the acetamido NH and carboxyls [5]. Depending on the conditions, such as temperature and pH, these H-bonds may be intramolecular or intermolecular. Although the CMHA used in our study is not high MW, there may still be an interplay between inter- and intramolecular interactions. In such case, our results suggest that the balance of inter- versus intramolecular H-bonding may depend on the number of carboxyls present, and the shift in balance leads to changes in the overall viscosity of the solution as the degree of modification for the CMHA changes. It would be interesting to synthesize CMHA with a substantially higher (or lower) MW to determine whether the same effects are observed in a different MW range.

Other researchers have also seen a decrease in viscosity as HA is modified. For example, it was found that HA with a 50% benzyl modification led to a decrease in steady-shear viscosity compared to native HA, although it was surmised that the decrease may be due to the decrease in hydrophilicity with added benzyl groups [33]. Recently, a report indicated that modifying HA with a short alkyl chain led to an increase in G’ up to about 3–4% modification; G’ then decreased with further increases in modification up to at least 11% modification [34], the upper limit in that study.

Another study also indicated that lower MW HA (350 kDa in that study, similar to CMHA here) in PBS is not as entangled as high MW HA, behaving as a viscous polymer fluid that is not cohesive [6]. Our results support this as well, particularly given that G” was always greater than G’ in the range tested (data not shown, see Materials & Methods), and the lack of shear thinning for even the mock CMHA.

Yet another study on solutions of HA in PBS suggested that the biomolecule simply behaves as a flexible polyelectrolyte in the high-salt limit and not as a reversible gel [35]. Our results are not inconsistent with this, given the concentration dependence of the viscosity of CMHA and HA solutions in PBS we observed, as well as the apparent increase in viscosity as the PBS concentration decreased and was no longer in the high-salt limit. It is interesting to note, however, that we also observed an increase in viscosity in the saline solutions compared to the 1X PBS for HA, mock CMHA, and high CM modification. Both the saline and 1X PBS are considered “high-salt limit” solutions, even for CMHA with 74% CM modification. These results may indicate an influence of the phosphates in the PBS samples. Despite the difference in viscosity between the mock CMHA in 1X PBS compared to saline, the conductivities are very similar, indicating that both ion type and concentration in the solvent affect the viscosity.

As previously mentioned, the pH of a solution may affect H-bonding between the acetamido and carboxyl groups. Gatej et al. [36] examined the effect of pH on solutions of HA in 0.15M NaCl and saw very little effect, except for a spike in viscosity at about pH 2.5. They attributed this to intermolecular bonding resulting from a change in the net charge of the polymer at this pH. The same spike in viscosity at pH 2.5 was seen in our study for a solution of mock CMHA in saline. However, this spike did not occur for the solution of CMHA in saline or DI water, and is therefore likely due to the additional charges introduced through the added carboxyls.

The results found in this study indicate that not only do the concentration and MW of HA affect solution viscosity, but so does the introduction of groups that alter the net charge of the biopolymer. This highlights the fact that multiple factors affect the physical properties for these large charged biopolymers in solutions, and therefore these factors must be taken into consideration when making modifications to the biopolymer. However, because these are naturally-derived polymers that are modified, and the process can alter not only degree of modification but MW as well, targeting a particular desired physical property may be more complicated than for synthetic polymers.

## Supporting Information

S1 Dataset Data used to create Figs 3–7. (XLSX)
