# Correction: Effect of Carboxymethylation on the Rheological Properties of Hyaluronan

**DOI:** 10.1371/journal.pone.0220964

**Published:** 2019-08-05

**Authors:** Rian J. Wendling, Amanda M. Christensen, Arthur D. Quast, Sarah K. Atzet, Brenda K. Mann

In the Determination of carboxyl modification subsection of the Materials and Methods, there is an error in the second equation. The numerator is missing the term *m*_*dry*_. Please view the complete, correction equation here:

$$DS=(MW_{DSU}\times n_{COOH}-m_{dry})÷(m_{dry}-MW_{I}\times n_{COOH})$$
